# Risk factors for mortality in patients with acute leukemia and bloodstream infections in the era of multiresistance

**DOI:** 10.1371/journal.pone.0199531

**Published:** 2018-06-28

**Authors:** Carolina Garcia-Vidal, Celia Cardozo-Espinola, Pedro Puerta-Alcalde, Francesc Marco, Adrian Tellez, Daiana Agüero, Francisco Romero-Santana, Marina Díaz-Beyá, Eva Giné, Laura Morata, Olga Rodríguez-Núñez, Jose Antonio Martinez, Josep Mensa, Jordi Esteve, Alex Soriano

**Affiliations:** 1 Infectious Diseases Department, Hospital Clínic-IDIBAPS, Barcelona, Spain; 2 Microbiology Department, Centre Diagnòstic Biomèdic. Hospital Clínic, Barcelona, Spain; 3 ISGlobal, Barcelona Center for International Health Research (CRESIB), Hospital Clínic-Universitat de Barcelona, Barcelona, Spain; 4 Internal Medicine Department, Hospital Insular Universitario de Gran Canaria, Las Palmas de Gran Canaria, España; 5 Hematology Department, Hospital Clínic-IDIBAPS, Barcelona, Spain; Azienda Ospedaliera Universitaria di Perugia, ITALY

## Abstract

**Objectives:**

We assess the epidemiology and risk factors for mortality of bloodstream infection (BSI) in patients with acute leukemia (AL).

**Methods:**

Prospectively collected data of a cohort study from July 2004 to February 2016. Multivariate analyses were performed.

**Results:**

589 episodes of BSI were documented in 357 AL patients, 55% caused by gram-positive bacteria (coagulase-negative staphylococci 35.7%, *Enterococcus* spp 10.8%) and 43.5% by gram-negative bacteria (*E*. *coli* 21%, PA 12%). We identified 110 (18.7%) multidrug-resistant (MDR) microorganisms, especially MDR-*Pseudomonas aeruginosa* (7%) and extended-spectrum beta-lactamase producing *Enterobacteriaceae* (7%). The 30-day mortality was 14.8%. Age (OR 3.1; 95% CI 1.7–5.7); chronic lung disease (4.8; 1.1–21.8); fatal prognosis according to McCabe index (13.9; 6.4–30.3); shock (3.8; 1.9–7.7); pulmonary infection (3.6; 1.3–9.9); and MDR-PA infections with inappropriate treatment (12.8; 4.1–40.5) were related to mortality. MDR-PA BSI was associated to prior antipseudomonal cephalosporin use (9.31; 4.38–19.79); current use of betalactams (2.01; 1.01–4.3); shock (2.63; 1.03–6.7) and pulmonary source of infection (9.6; 3.4–27.21).

**Conclusions:**

MDR organisms were commonly isolated in BSI in AL. Inappropriate empiric antibiotic treatment for MDR-PA is the primary factor related to mortality that can be changed. New treatment strategies to improve the coverage of MDR-PA BSI should be considered in those patients with risk factors for this infection.

## Introduction

Bloodstream infections (BSI) are frequently observed in patients with haematological malignancies with a prevalence that ranges from 11% to 38% [1–5]. Crude mortality rates vary from 12% to 42%, and attributable mortality rates as high as 30% have been reported in some studies [4–6]. Among patients with haematological malignancies, patients with acute leukemia (AL) are a unique cohort. From AL diagnosis, patients undergo in a process with continuous cycles of chemotherapy and profound prolonged neutropenia. Moreover, integrity alteration of the gastrointestinal mucosa, and widespread use of indwelling intravascular catheters cause that these patients are a high-risk group for BSI [1,3–5]. High consumption of antibiotics and prolonged hospitalizations might render these patients vulnerable to being colonized by multidrug-resistant (MDR) strains. Epidemiology of BSI in this population is in a continuous shift due to changes in chemotherapies, immunosuppressive agents, and prophylaxis strategies.

However, in this era of problematic infections caused by MDR-bacteria, limited data have been previously reported on epidemiology, outcomes and risk factors for mortality of BSI in this specific patient group. This information is crucial for improving the empirical treatment of patients and the potential antibiotic prophylaxis strategies. The knowledge of this epidemiology should also be taken into account in the management of febrile neutropenia in AL patients.

We aimed to describe the current epidemiology and its changes during the different cycles of chemotherapy of BSI in a large current cohort of patients with AL. We also assessed the risk factors for BSI mortality and the risk factors for BSI caused by MDR-*Pseudomonas aeruginosa* (MDR-PA).

## Materials and methods

### Setting and data collection

This study was performed at the Hospital Clinic in Barcelona (Spain), a 700-bed university center that provides specialized and broad medical, surgical, and intensive care for an urban population of 500,000 people.

Since 1991 our institution has carried out a blood culture surveillance program identifying and monitoring all patients with bacteraemia. The collected data were entered in a specific database designed for this program. Patients were prospectively followed up 30 days after onset, by a senior infectious disease specialist: patient’s medical history, physical examination, the results of other microbiological tests and complementary imaging explorations were assessed in order to determine the source of infection and recommend appropriate antibiotic treatment.

### Study population and design

For this study, we analysed all consecutive episodes of BSI occurring in patients with AL from July 2004 to February 2016.

The following data were obtained from all patients: age, gender, comorbidities, McCabe classification of underlying diseases, treatment with antibiotics or steroids in the previous month, recent hospitalization (within the last month), surgery and other invasive procedures, presence of central venous catheter or urinary catheter, radiological findings, current administration of anti-neoplastic chemotherapy, leucocyte count, source of bacteraemia, length of hospitalization before diagnosis of BSI, days of neutropenia before BSI, mucositis, fever and shock on presentation, intensive care unit and need for mechanical ventilation, etiologic microorganisms and their susceptibility profile, empirical antibiotic treatment, appropriateness of empirical therapy, definitive antibiotic therapy, and early and overall mortality.

The study was approved by the Ethics Committee board (Comité Ético de Investigación Clínica del Hospital Clínic de Barcelona) of our institution.

### Definitions

Neutropenia was defined as an absolute neutrophil count of <500 cells/mm3. Mucositis was defined following the WHO criteria [7]. Prognosis of the underlying disease was classified, according to McCabe and Jackson modified criteria, as rapidly fatal (death expected within 3 months), ultimately fatal (death expected within a period of >3 months but <5 years) and non-fatal (life expectancy >5 years). Shock was defined as having a systolic pressure <90 mmHg that was unresponsive to fluid treatment or required vasoactive drug therapy. Prior antibiotic therapy was defined as the use of any antimicrobial agent for ≥3 days during the month prior to the occurrence of the bacteraemic episode. According to the protocols of our hospital, patients with an expected neutropenia over 10 days received prophylaxis with a fluoroquinolone. Definitions of healthcare-associated and community-acquired BSI as well as definition of the source of infection have been previously provided [8].

The following gram-negative bacilli were considered to be MDR: 1) extended-spectrum beta-lactamase (ESBL) producing *Enterobacteriaceae*, 2) AmpC cephalosporinase hyperproducing *Enterobacteriaceae*, 3) MDR strains of non-fermenting GNB such as *Pseudomonas aeruginosa*, *Acinetobacter baumannii* and *Stenotrophomonas maltophilia*. Non-fermenting GNB were defined as MDR strains when they were resistant to at least 3 classes of antibiotics: carbapenems, ureidopenicillins, cephalosporins (ceftazidime and cefepime), monobactams, aminoglycosides, and fluoroquinolones. Extensively drug-resistant (XDR) GNB were those non-susceptible to ≥1 agent in all but ≤2 categories. MDR gram-positive organisms included methicillin-resistant *Staphylococcus aureus* (MRSA) and vancomycin-resistant *Enterococcus faecium* (VRE).

Appropriate empirical therapy was considered when the patient received at least one in vitro active antimicrobial agent within 24 h after obtaining blood cultures before susceptibility results were available, and the dosage and route of administration were in accordance with current medical standards. Early mortality was defined as death within 48 h of the onset of BSI and overall mortality as death by any cause within the first 30 days of onset. Death was considered related to the BSI if it occurred before the resolution of symptoms or signs, or within 7 days of the onset of bacteraemia, and there was no other explanation.

### Microbiological methods

Blood samples were processed using the BACTEC 9240 system or Bactec FX system (Becton-Dickinson Microbiology Systems), with an incubation period of 5 days. Isolates were identified by standard techniques. Antimicrobial susceptibility testing was performed by using a microdilution system (Microscan WalkAway Dade Behring, West Sacramento, CA or Phoenix system, Becton Dickinson, Franklin Lakes, NJ) or the Etest (AB Biodisk, Solna, Sweden/ bioMérieux, Mercy l’Etoile, France). Current Clinical and Laboratory Standards Institute (CLSI) or EUCAST breakpoints for each year were used to define susceptibility or resistance to these antimicrobial agents, and intermediate susceptibility was considered as resistance. All MDR strains were confirmed by e-test methods over the study period.

### Statistical analysis

Categorical variables were compared by Chi-square or Fisher’s exact test when necessary and Student’s t-test or the Mann–Whitney U-test for continuous variables. Chi-square for trend analysis was conducted to compare changes in epidemiology over time. Two multivariate regression models (step-forward procedure) were used to identify the independent risk factors for MDR-PA and the independent risk factors for overall mortality, respectively. The goodness of fit of the multivariate models was assessed by the Hosmer-Lemeshow test and the area under the receiver operating characteristic curve. The threshold for statistical significance was defined as a two-tailed p<0.05. All analyses were done by using the SPSS software (version 18.0; SPSS, Inc., Chicago, IL).

## Results

### Demographics and epidemiology

In total, 589 BSI episodes of bloodstream infection in 357 patients with AL were documented within the study period. Table 1 summarizes the demographic and clinical characteristics of the patients. Most of the BSIs were nosocomially (52.6%) acquired. Three hundred and sixty-two (61.5%) patients had received previous antibiotic therapy, mainly quinolones (34.5%), cephalosporins (34.3%), and carbapenems (22.6%).

**Table 1:** Demographic and clinical characteristics of 589 BSI episodes in patients with acute leukemia.

	Episodes N = 589 (%)
**Demographics**	
** Male sex**	324 (55)
** Age, median (IQR) years**	53 (40.5–64)
**Comorbid conditions**	
** Diabetes mellitus**	37 (6.3)
COPD*	12 (2)
**Clinical conditions**	
** Previous hospital admission (last month)**	254 (43)
** Previous antibiotic therapy (last month)**	362 (61.5)
** Central intravenous vascular catheter**	479 (81.3)
** Urinary catheter**	40 (6.8)
** Corticosteroid therapy**	204 (34.5)
** Neutropenia (<500 neutrophils)**	374 (63.5)
** Days of neutropenia before BSI, median (IQR) days**	3 (0–10)
** Mucositis**	101 (17.5)
**Site of acquisition**	
** Nosocomial**	310 (52.6)
** Healthcare**	244 (41.4)
** Community**	28 (4.8)
**Source of bacteraemia**	
** Unknown origin**	294 (49.9)
** Catheter-related**	211 (35.8)
** Pneumonia**	27 (4.6)
** Urinary tract**	17 (2.9)
** Abdominal**	14 (2.4)
** Skin and soft tissue infection**	12 (2)
** Cholangitis**	1 (0.2)

**IQR**: Interquartile range.

*COPD: Chronic Obstructive Pulmonary Disease.

Table 2 details organisms responsible for all episodes of BSI. Gram-positive organisms accounted for 55% of cases. The most frequent gram-positive isolated were coagulase-negative staphylococci (35.7%), followed by *Enterococcus* spp (10.8%). Among gram-negative organisms (43.5%), *Escherichia coli* (20.5%) was the most frequently isolated, followed by *Pseudomonas aeruginosa* (12%). There were 25 (4%) episodes of candidemia. Forty-eight BSI episodes were polymicrobial (8%). Patients with oral mucositis had more commonly candidemia (10.9% vs 2.9%; p<0.001) and less commonly gram-negative bacteremia (34.7% vs 45.1%; p = 0.055). No significant differences were found in gram-positive bacteremia (55.4% vs 54.9%; p = 0.924).

**Table 2:** Detected microorganisms during different chemotherapy cycles.

	Total N = 589 (%)	Induction N = 103 (%)	Consolidation N = 176 (%)	Neutropenia N = 376
**Gram negative-bacteria**	257 (43.6)	39 (37.9)	75 (42.6)	82 (39)
***E*. *coli***	120 (20.4)	13 (12.6)	40 (22.7)	95 (25.3)
Ciprofloxacin R*	99 (82.5)	11 (84.6)	37 (92.5)	81 (85.3)
ESBL*	31 (25.8)	2 (15.4)	9 (5.1)	24 (25.2)
***Klebsiella* spp**	28 (4.8)	5 (4.9)	7 (4)	15 (4)
Ciprofloxacin R*	13 (46.4)	4 (80)	5 (71.4)	9 (60)
ESBL*	10 (35.7)	4 (80)	4 (2.3)	7 (46.6)
***P*. *aeruginosa***	71 (12)	12 (11.7)	23 (13.1)	43 (11.4)
Ciprofloxacin R*	42 (59.2)	8 (66.7)	15 (65.2)	26 (60.5)
MDR*	41 (57.7)	8 (66.7)	15 (65.2)	25 (58.1)
***S*. *maltophilia***	12 (2.0)	6 (5.8)	1 (0.6)	9 (2.4)
***Enterobacter* spp**	8 (1.4)	1	1 (0.6)	4 (1.1)
**Gram positive-bacteria**	323 (55)	54 (52.4)	102 (58.0)	127 (60.5)
***S*. *aureus***	19 (3.2)	3 (2.9)	6 (3.4)	7 (1.9)
MRSA*	4 (21)	0	0	1(0.3)
** CoNS**	211 (35.8)	34 (33)	76 (43.2)	135(35.9)
***Enterococcus* spp**	64 (10.8)	12 (11.7)	9 (5.1)	37(9.8)
*E*. faecalis*	29 (45.3)	2 (16.7)	5 (55.6)	11 (29.7)
*E*. faecium*	33 (51.6)	10 (83.3)	4 (44.4)	21 (56.8)
VRE*	0	0	0	0
***S*. *pneumoniae***	6 (1)	0	1 (0.6)	0
** Group viridans streptococci**	18 (3)	5 (4.9)	7 (4)	11(2.9)
***L*. *monocytogenes***	6 (1)	1 (1)	1 (0.6)	2(0.5)
***Candida***	25 (4.2)	13 (12.6)	4 (2.3)	19(5.1)
**Polymicrobial**	57 (9.6)	8 (7.8)	13 (7.4)	27(7.2)
**MDR isolates**	110 (18.7)	21 (20.4)	31 (17.6)	73 (19.5)

*Percentage among their species.

We identified 110 (18.7%) MDR organisms, mostly MDR-PA (6.9%) and ESBL-producing *E*. *coli* and *Klebsiella* spp (6.9%). No vancomycin-resistant *Enterococcus* spp or carbapenemase-producing *Enterobacteriaceae* were found. We did not identify any special temporal trend in resistance or in causative BSI microorganisms during different chemotherapy cycles (Table 2). Table 3 details epidemiological changes over time during the study period. No differences in MDR-GNB or MDR-PA were found.

**Table 3:** Changes over time in epidemiology of BSI in HSCT recipients.

	2004–2007 N = 233 (%)	2008–2011 N = 215 (%)	2012–2016 N = 141 (%)	p-value for trend
**Gram-positive bacteria**	137 (58.8)	113 (52.6)	73 (51.8)	0.151
**CoNS**	97 (41.6)	74 (34.4)	40 (28.4)	0.008
***S*. *aureus***	10 (4.3)	6 (2.8)	3 (2.1)	0.229
** MRSA**	2 (0.9)	2 (0.9)	0	0.378
***Enterococcus* spp**	16 (6.9)	25 (11.6)	23 (16.3)	0.004
**Gram-negative bacteria**	92 (39.5)	103 (47.9)	60 (42.6)	0.405
***E*. *coli***	54 (23.2)	39 (18.1)	27 (19.1)	0.280
** ESBL**	15 (6.4)	8 (3.7)	8 (5.7)	0.611
***P*. *aeruginosa***	19 (8.2)	35 (16.3)	17 (12.1)	0.141
** MDR**	10 (4.3)	20 (9.3)	11 (7.8)	0.125
***Klebsiella* spp**	8 (3.4)	13 (6)	7 (5)	0.404
** ESBL**	6 (2.6)	3 (1.4)	1 (0.7)	0.161
**MDR isolates**	43 (18.5)	44 (20.5)	23 (16.3)	0.703
**Candidemia**	10 (4.3)	3 (1.4)	12 (8.5)	0.123

### Therapeutic approaches and outcomes

Most patients received empirical antibiotics (96.8%), the most prevalent were carbapenems (57%), glycopeptides (50.6%) and aminoglycosides (25.6%). Inadequate empirical antibiotic therapy was given to 26.3% of patients. Table 4 summarizes the etiology of BSI in patients who received inadequate empirical antibiotic treatment. Those patients with BSI due to a MDR strain had a higher percentage of initial inadequate treatment than patients without MDR strains (35.5% vs 24.4% p = 0.018).

**Table 4:** Most frequent microbiological isolates in those 155 BSI episodes who received inappropriate antibiotic therapy.

Microorganism	Inadequate initial empirical therapy N = 155 (%)
**Gram negative-bacteria**	
***E*. *coli***	16 (10.3)
ESBL*	10 (62.5)
***Pseudomonas***	21 (13.5)
MDR*	19 (90.5)
***S*. *maltophilia***	9 (5.8)
**Gram positive-bacteria**	
** CoNS**	65 (41.9)
***Enterococcus* spp**	22 (14.2)
*E*. faecium*	14 (63.6)
***Candida* spp**	16 (10.3)

*Percentage pertaining to their species.

Shock was documented in 11.2% of cases. Early mortality, overall mortality, and related mortality within the first 30 days were 3.7%, 14.8% and 8.7%, respectively. In episodes caused by MDR organisms the rates were 4.5%, 19.1% and 12.7%. In comparison to patients without MDR organisms, those with MDR organisms who received inappropriate antibiotic treatment had higher early mortality (10.3%, p = 0.05), overall mortality (33.3%; p = 0.005) and related mortality (20.5; p = 0.069), especially those with MDR-PA infection (early (15.8%, p = 0.03), 30-days (63.2%, p< 0.001) and related (36.8%, p< 0.001) mortality).

### Predictors of mortality

Table 5 detailed the univariate analysis for mortality. Table 6 showed the multivariate analysis of predictors for overall mortality. Age (OR 3.1; 95% CI 1.7–5.7); chronic lung disease (4.8; 1.1–21.8); fatal prognosis according to McCabe index (13.9; 6.4–30.3); shock at onset (3.8; 1.9–7.7); pulmonary source of infection (3.6; 1.3–9.9); and BSI caused by MDR-PA with inappropriate antibiotic therapy (12.8; 4.1–40.5) were independent risk factors for overall mortality. The goodness of fit of the multivariate model was assessed by the Hosmer-Lemeshow test (0.784), and the discriminatory power of the score, as evaluated by the area under the receiver operating characteristic curve, was 0.848 (95% 0.797–0.900), demonstrating a strong ability to predict overall mortality in patients with AL and BSI.

**Table 5:** Univariate analysis for mortality.

	Deaths N = 87 (%)	Survivors N = 502 (%)	*p* value
**Clinical characteristics**			
** Male sex**	45 (51.7)	279 (55.7)	0.493
** Older age (>65 years old)**	47 (54)	95 (18.9)	< 0.001
** Diabetes mellitus**	11 (12.6)	26 (5.2)	0.008
** Chronic lung disease**	5 (5.7)	7 (1.4)	0.008
** Chronic liver disease**	2 (2.3)	4 (0.8)	0.218
** Corticosteroid therapy**	36 (41.4)	167 (33.3)	0.151
** Neutropenia (<500 neutrophils)**	47 (54)	327 (65.1)	0.069
** Prolonged neutropenia (more than 21 days)**	18 (20.7)	55 (11)	0.011
** Mucositis**	6 (6.9)	95 (18.9)	0.006
** BSI in the first month after AL diagnosis**	19 (21.8)	80 (15.9)	0.177
** AL in induction treatment**	15 (17.2)	88 (17.5)	0.948
** Fatal prognosis according to McCabe index**	29 (33.3)	16 (3.2)	< 0.001
**Site of acquisition**			
** Nosocomial**	43 (49.4)	267 (53.2)	0.547
** Community**	9 (10.3)	19 (3.8)	0.008
**Source of bacteraemia**			
** Unknown origin**	38 (43.7)	256 (51)	0.208
** Catheter-related**	22 (25.3)	189 (37.6)	0.026
** Pulmonary source of infection**	13 (14.9)	14 (2.8)	< 0.001
** Urinary tract**	5 (5.7)	12 (2.4)	0.084
** Intraabdominal source of infection**	5 (5.7)	9 (1.8)	0.025
**Etiology**			
***S*. *aureus***	2 (2.3)	17 (3.4)	0.596
***CoNS***	12 (13.8)	119 (23.7)	< 0.001
** Group viridans streptococci**	0 (0)	18 (3.6)	0.091
***Enterococcus* spp**	15 (17.2)	49 (9.8)	0.038
***E*. *coli***	17 (19.5)	103 (20.5)	0.834
***P*. *aeruginosa***	24 (27.6)	47 (9.4)	< 0.001
***Klebsiella* spp**	7 (8)	21 (4.2)	0.118
***Candida* spp**	5 (5.7)	20 (4)	0.451
** Polymicrobial**	9 (10.3)	39 (7.8)	0.418
** MDR strain**	21 (24.1)	89 (17.7)	0.157
**Inappropriate antibiotic therapy**			
**Clinical and treatment features**			
** Shock at onset**	23 (26.4)	43 (8.6)	< 0.001
** Persistent bacteremia**	12 (13.8)	85 (16.9)	0.486
** Inappropriate antibiotic therapy**	30 (34.5)	125 (24.9)	0.055
** Inappropriate antibiotic therapy in MDR strains**	13 (14.9)	26 (5.2)	0.001
** Inappropriate antibiotic therapy in MDR-PA**	12 (13.8)	7 (1.4)	< 0.001

**Table 6:** Independent risk factors for mortality.

*Risk factor*	*Adjusted odds ratio (95% CI)*	*p-value*
Older age (>65 years old)	3.116 (1.698–5.719)	<0.001
Chronic lung disease	4.890 (1.096–21.818)	**0.033**
Fatal prognosis according to McCabe index	13.89 (6.373–30.291)	**<0.001**
Pulmonary source of infection	3.582 (1.296–9.903)	0.014
Shock at onset	3.789 (1.865–7.698)	**<0.001**
BSI caused by MDR-PA with inappropriate antibiotic therapy	12.818 (4.056–40.502)	<0.001

Adjusted for: mucositis, diabetes, communitary-infection, intrabdominal source of infection, catheter source of infection, prolonged neutropenia (more than 21 days), BSI caused by *Enteroccocus* spp, inappropriate antibiotic treatment, and BSI caused by MDR-bacteria with inappropriate antibiotic therapy.

### Risk factors for BSI caused by MDR-PA

Independent factors associated with the isolation of MDR-PA in BSI in our cohort of patients with AL were prior antipseudomonal cephalosporin use (OR 9.31; 95% CI 4.38–19.79); bloodstream infection occurring within betalactam antibiotic therapy (2.01; 1.01–4.3); shock at onset (2.63; 1.03–6.7) and pulmonary source of infection (9.6; 3.4–27.21). The goodness of fit of the multivariate model was assessed by the Hosmer-Lemeshow test (0.658), and the discriminatory power of the score, as evaluated by the area under the receiver operating characteristic curve, was 0.807 (95% 0.724–0.890), demonstrating a strong ability to predict MDR-PA BSI.

## Discussion

This prospective study describes the epidemiology and prognostic factors of a current cohort of patients with AL presenting with a BSI. The most important findings were: 1) the proportion of gram-positive and gram-negative bacteria isolated was similar 2) the causative microorganisms did not change significantly between the different chemotherapy cycles 3) the presence of MDR microorganisms, mainly GNB, was common 4) inadequate empirical antibiotic treatment was frequent, especially in patients with MDR BSI 5) the overall mortality was high 6) independent risk factors for mortality were older age, chronic lung disease, fatal prognosis according to McCabe index, shock at onset, pulmonary source of infection and BSI caused by MDR-PA with inappropriate antibiotic therapy 7) independent risk factors for MDR-PA BSI were prior antipseudomonal cephalosporin use, current use of betalactams, shock at onset, and pulmonary source of infection.

The epidemiology of BSI in patients with AL is in a continuous shift [4,5,9,10]. Our current data showed a slight predominance of Gram-positive organisms, mainly CoNS and enterococci. Moreover, we documented an important number of patients with BSI caused by Gram-negative bacilli. These results are similar to those obtained in comparable centers in overall haematological patients [11–14].

The development of multidrug resistance has become a major health problem worldwide [15–16]. Information dealing with the current epidemiology and outcomes of MDR-bacteria in patients with haematological malignancies is scarce [6,17,18], and there specifically lacks data in the AL population. We found a high proportion of MDR isolates, predominantly MDR-PA and ESBL producing *E*. *coli* and *K*. *pneumoniae*. Among Gram-positive organisms, resistance to methicillin in *S*. *aureus* isolates was relatively low (0.7%) in this population, and as expected in our geographical area no vancomycin-resistant *Enterococcus* were detected [19,20]. Our study confirms that a high number of patients with MDR-BSI receive an inappropriate empirical treatment. Remarkably, we did not find differences in global microbiology and MDR isolates according to the different chemotherapy phases. The finding that antibiotic resistance did not change during the progressive cycles of therapy might be explained by the fact that initial induction chemotherapy is the most aggressive cycle of chemotherapy; always administered at hospital; with the host in the weakest state, and with a high antibiotic pressure.

In the present study, we found early and overall mortality rates of 3.7% and 14.8% respectively, with a related mortality of 8.7%. These findings are consistent with other studies involving patients with haematological malignancies [4–6,12,21]. We identify some factors related with overall mortality (older age, chronic lung disease, fatal prognosis according to McCabe index, shock at onset, pulmonary source of infection) that concords with previous research conducted mainly in patients with overall haematological malignances [22–25]. Also, we identify that inadequate empirical therapy for MDR-PA BSI was associated with an 11-fold increase in mortality. This factor is important because it can be modified.

The incidence of MDR-PA has recently increased worldwide [26,27]. The impact of inappropriate empirical antibiotic therapy in mortality of BSI caused by MDR-PA has been a matter of debate for many years with conflicting results [28–30]. The discrepancies among studies reflect the complexity of this infection and the key role that antibiotic therapy not only plays, but as well as host factors, source of infection or removal of infection site. However, to our best knowledge, specific studies on the impact of inappropriate antibiotic therapy in BSI caused by MDR-PA in AL population have not been previously reported.

Improving the recognition of patients with AL and BSI at risk of MDR-PA is mandatory. We identified that those patients with prior antipseudomonal cephalosporin use, those who had current use of betalactamics, and those with shock at onset or/and pulmonary source of infection had a high risk for BSI caused by MDR-PA. Surveillance data of the resistance mechanisms of PA in the geographical area or institution may help to choose the best optimal antibiotic regimen. Whether the screening of MDR-strains colonization might help to diminish inappropriate empirical antibiotic therapy remains a matter of debate [31,32]. Initial approach of these patients with an empirical antibiotic therapy comprising of a combination of a carbapenem in continuous infusion, with either an aminoglycoside or a different antipseudomonal approach including ceftolozane/tazobactam, might be suitable options.

The strengths of this study are the large number of patients included, the prospective collection of the data, and the comprehensive clinical and microbiologic data gathered. However, there are some limitations that should be acknowledged. Our study was conducted at a single center; considering that different microbiological epidemiology varies widely from hospital to hospital and even in different wards of the same hospital, it is difficult to generalize some conclusions drawn. Furthermore, we perform quinolone prophylaxis for patients expecting to have a neutropenia longer than 10 days, making our results perhaps different from those other centers who do not perform prophylaxis.

In conclusion, we found a similar proportion of Gram-positive and Gram-negative organisms producing BSI in patients with AL, without changes according to the chemotherapy phase. MDR microorganisms were frequent and commonly received inadequate empirical antibiotic therapy. Mortality in patients with AL and BSI remains high, especially in older patients with poor baseline prognosis, and in patients with severe infection at onset. Improving empirical antibiotic treatment, especially for patients with BSI caused by MDR-PA, is needed. There must be a high suspicion of infection caused by MDR-PA in patients presenting with prior antipseudomonal cephalosporin use, current use of betalactamics at BSI onset, shock, or pulmonary source of infection.

## Supporting information

S1 File (SAV)
